# Heat Stroke: A Medical Emergency Appearing in New Regions

**DOI:** 10.1155/2017/6219236

**Published:** 2017-09-13

**Authors:** Sofie Søndergaard Mørch, Johnny Dohn Holmgren Andersen, Morten Heiberg Bestle

**Affiliations:** ^1^Department of Anaesthesiology and Intensive Care, Bispebjerg Hospital, Bispebjerg Bakke 23, 2400 København NV, Denmark; ^2^Department of Anaesthesiology and Intensive Care, Hvidovre Hospital, Kettegård Alle 30, 2650 Hvidovre, Denmark; ^3^Department of Anaesthesiology and Intensive Care, Nordsjællands Hospital, Dyrehavevej 29, 3400 Hillerød, Denmark

## Abstract

Heat stroke is an acute, life-threatening emergency characterized clinically by elevated body temperature and central nervous system dysfunction. Early recognition and treatment including aggressive cooling and management of life-threatening systemic complications are essential to reduce morbidity and mortality. This case report describes two Danish patients diagnosed with heat stroke syndrome during a heat wave in the summer of 2014. Both patients were morbidly obese and had several predisposing illnesses. However since heat stroke is a rare condition in areas with temperate climate, they were not diagnosed until several days after admittance; hence treatment with cooling was delayed. Both patients were admitted to the intensive care unit, where they were treated with an external cooling device and received treatment for complications. Both cases ended fatally. As global warming continues, more heat waves will occur in previously cooler regions. Therefore it is important to raise awareness of heat stroke since outcome depends on early recognition and rapid cooling.

## 1. Introduction

Classic heat stroke (HS) is an unusual diagnosis in countries with temperate climate. However, as global warming has resulted in a higher frequency of heat waves, an increase in weather-related heat deaths is seen [1–5].

Heat illnesses cover a spectrum of syndromes culminating with the acute, life-threatening emergency of HS that carries a high mortality [2, 3, 6–12]. HS is traditionally defined as a high core temperature accompanied by encephalopathy [6, 10–13]. An alternative definition that takes into consideration the pathophysiology is gaining recognition. This defines HS as hyperthermia associated with a systemic inflammatory response leading to multiorgan dysfunction in which encephalopathy predominates [11, 14].

Two forms of HS are recognized. Exertional HS primarily affects young and healthy athletic patients exercising vigorously in hot and humid climates until the body's normal thermoregulatory mechanisms are overwhelmed. Exertional HS is characterized by rapid onset and is frequently associated with a high core temperature [2, 3, 6, 7, 9, 10, 12, 13, 15].

Classic (passive or nonexertional) HS is caused by environmental exposure and occurs in young children, in elderly patients or in patients with underlying chronic illnesses, who are exposed to extreme environmental conditions. Classic HS can develop slowly over several days [2, 3, 7, 9, 10, 12, 13, 15].

HS occurs when the body ceases to dissipate heat adequately because of extreme environmental conditions or increased endogenous heat production [15]. When the body's thermoregulatory mechanisms are exceeded and fail, a variable degree of organ failure may occur [15]. Furthermore complex immunological and inflammatory processes that resemble SIRS (systemic inflammatory response syndrome) contribute to the illness [2, 3, 6, 10–14]. Rapid diagnosis and prompt cooling are pivotal, since the condition triggers a cascade of metabolic events that may progress to irreversible injury or death [2, 4, 6, 13]. In-hospital mortality for classical HS ranges from 10 to 65% [2].

This case report describes two Danish patients suffering from HS that was initially overlooked at hospital admission. Both patients report one week's malaise, elevated temperature, and symptoms suggesting cerebral dysfunction.

Written informed consent was obtained from relatives for publication of this case report.

## 2. Case Presentation

*Patient 1* was a 64-year-old woman with a premedical history of unspecified mental disorder, musculoskeletal pain, and severe obesity (145 kg, BMI 57). She appeared neglected and was described as tending to isolate herself at home.

At the hospital admission she presented with Glasgow Coma Score (GCS) 5-6, hypotension, tachycardia, and divergent, fixed dilated pupils. Arterial blood gas analyses showed metabolic acidosis (pH 7.15, lactate 4.6 mmol/L, BE −11 mmol/L). Sepsis was suspected on the background of aspiration pneumonia and treatment was initiated with antibiotics and fluid replacement, soon after she was intubated and transferred to the intensive care unit (ICU).

Primary findings in the ICU were GCS 3, tachycardia (130 beats/minute), tachypnoea, hyperthermia (39.9°C), and decreased urine output. The initial wide panel of blood tests were normal except INR 1.3, potassium 2.9 mmol/L, glucose 13.3 mmol/L, troponin T 10300 ng/L, and a normal CK-MB. Nasogastric aspiration contained coffee-grounds and a urine test strip revealed presence of blood, protein, nitrite, and glucose. A neurological evaluation and a head CT-scan were performed; both were normal.

During the first days after admission a large urine output, hyperthermia, and setting sun eye phenomenon were observed. Oxygen demand increased and cooling with ice and fanning were initiated. MRI, lumbar puncture, and EEG were all normal. Microbiological tests showed no sign of infection.

The fifth day after ICU admission, HS syndrome was suspected and active cooling was intensified using a thermoregulatory device with cooling pads. The patient started to recover and the dose of cardiac inotropes was gradually reduced and then ceased. For the first time since admission some levels of contact were described. An infection with* Staphylococcus* bacteria halts improvement and her level of consciousness fell. A neurologic examination revealed an abnormal EEG and positive Babinski sign. On day 21 a PET-CT was conducted, showing globally reduced, cortical metabolic activity. It was therefore decided not to escalate treatment. The patient was extubated and died 34 days after hospital admission.

*Patient 2* was a 74-year-old man suffering from heart failure, chronic atrial fibrillation, severe venous insufficiency, leg ulcers, type 2 diabetes, chronic obstructive lung disease, and severe obesity (160 kg, BMI 49). At hospital admission the patient was confused with delayed response time. Furthermore he presented with atrial fibrillation (140 beats/minute), hyperthermia (39.0°C), and elevated blood levels of creatinine. He was tachypnoeic with low oxygen saturation (91%). A urine test strip showed presence of blood, protein, and ketone bodies. Initial treatment included fluid therapy and antibiotics on the suspicion of sepsis. The patient was transferred to telemetry in the cardiology department where treatment with digoxin was initiated.

During the following days urine output increased, but his level of consciousness decreased and he developed myoclonia. Due to accumulation of carbon dioxide he was transferred to the ICU, where intubation and mechanical ventilation were initiated. At admission GCS score was 3, pupil reactions were sluggish, and muscle spasms in the upper body were observed. Blood tests were normal except an increasing INR.

During the first days in the ICU the patient improved with decreasing demand of oxygen and cardiac inotropes. Analysis of the cerebrospinal fluid and a head CT were both normal. Microbiological tests showed no sign of infection. Despite fanning and cold fluids the patient still had an elevated temperature and cooling was intensified using a thermoregulatory device with cooling pads. On day 5 the patient's temperature normalized and he gained consciousness. On day 7 the diagnosis of HS syndrome was suspected. The patient stabilized and was extubated, but he still received intermittent cooling. The patient developed hypercapnia and was reintubated and later a tracheostomy was performed. Due to anuria dialysis was performed until he regained his kidney function on day 23. The patient was again stabile and interacting with family and staff. Unfortunately the patient was infected with pneumonia. His overall condition thereafter gradually declines and he died 55 days after admission.

## 3. Discussion

Several factors predispose to the development of HS (Table 1). Both patients described above were morbidly obese, which is a risk factor for HS due to more insulation and a lower surface area-to-volume ratio resulting in less capacity for dissipating heat [8]. Furthermore Patient 1 had an unspecified mental disorder and tended to isolate herself in her home. Patient 2 suffered from cardiovascular disease, diabetes, and COLD.

Both patients presented with the two cardinal features of HS: hyperthermia and central nervous system dysfunction, although they were both faulty diagnosed at the hospital admittance.

The reason can be that HS is a systemic disorder that can mimic other medical conditions such as meningitis, encephalitis and septic shock, and myocardial infarction [2, 9]. These differential diagnoses were all examined and tested but excluded as test results came back normal. The patients were treated symptomatically since HS was not suspected until several days after admittance.

Contributing to the delayed diagnosis is the fact that classical HS is a very unusual cause of hyperthermia in temperate regions. But accompanying the rise in average global temperature is a rise in the frequency, intensity, and duration of heat waves and thereby weather-related deaths [3–5]. An increased awareness of HS is necessary not only in warmer regions, where the increased frequency and severity of heat waves probably will result in more cases, but also in regions where this HS was previously an unusual diagnosis due to cooler climate and few heat waves.

HS is a medical emergency and it is essential that doctors recognize the signs and initiate treatment rapidly to reduce morbidity and increase survival. Decreasing the core temperature below 38.9°C within 30 min of presentation significantly improves survival [3, 12]. In this case cooling was not initiated until more than 24 hours after arrival at the hospital and this may have influenced outcome. On the contrary, the delayed cooling may instead have exacerbated the conditions of the patients: elevated temperature several days after debut of the syndrome is more likely due to fever than hyperthermia. Since fever represents an increase in the set-point, cooling was probably ineffective and may even have aggravated the condition.

## 4. Conclusion

These cases demonstrate the importance of keeping heat related diseases in mind when ambient temperatures rise above normal. HS is a very rare diagnosis in Northern Europe and this is probably the main reason for the delayed diagnoses which might have contributed to the fatality of the cases. As climate models predict an increased frequency and severity of heat waves the incidence of heat stroke is expected to rise.

It is our hope that this case report will increase the awareness of heat stroke in all temperate areas of the world, since more cases may appear as the temperature rises.

## Figures and Tables

**Table 1:** Predisposing factors to classic heat stroke [1–3, 6, 8–15].

Age	Young children and individuals over 65 years

Diseases	Dermatologic conditions, infection, and endocrine disorders that increase endogenous heat production, cardiovascular disease, obesity, dehydration, pulmonary disease, neurologic disease, psychiatric illness, and previous history of heat-related illnesses^1^

Medicine	Beta-blockers, diuretics, calcium channel blockers, laxatives, anticholinergic drugs, salicylates, thyroid agonists, benzatropine, trifluoperazine, ephedra, certain diet pills, butyrophenones, alpha agonists, inhaled anaesthetics, monoamine oxidase inhibitors, and sympathomimetic medications

Drugs	Alcohol, cocaine, amphetamine, and derivatives, PCP and LSD

Social	Immobilization, isolation, being unable to care for oneself, low socioeconomic status, residing in upper floors in tall buildings, no air-condition at home, and being unable to secure a cooler environment for a few hours daily

Exposure	Prolonged sun exposure, recent move from a temperate to a hot climate, hot environments, and wearing excessive or protective clothing limiting heat dissipation

^1^Previous history of heat related illness is suspected to be a predisposing factor.
